# Crayfish bury their own exuviae: a newly discovered behavioral pattern in decapods

**DOI:** 10.1186/s40064-016-3343-6

**Published:** 2016-09-29

**Authors:** Miloš Buřič, Martin Fořt, Martin Bláha, Lukáš Veselý, Pavel Kozák, Antonín Kouba

**Affiliations:** Faculty of Fisheries and Protection of Waters, South Bohemian Research Center of Aquaculture and Biodiversity of Hydrocenoses, University of South Bohemia in Ceske Budejovice, Vodňany, Czech Republic

**Keywords:** Invertebrate, Behaviour, Hoarding, Caching, Crustacean, Crayfish

## Abstract

Invertebrates are a very diverse group of animals, showing a wide spectrum of life strategies and adaptations. They often exhibit very complex behavioural and social patterns. In crayfish, the largest freshwater invertebrates, we found a new behavioural pattern, burying their own exuviae after moulting. Such a pattern may be an as yet unrecognized type of hoarding or caching. The buried exuvia is exhumed after 2 or 3 days (when the crayfish body is no longer as soft) and consumed. This behaviour is probably self-protective (hiding the mark of a helpless prey), as well as having mineral storage reasons. Such complex behavioural patterns in invertebrates present new challenges for future research.

## Background

Caching or hoarding behaviour is well known, particularly in mammals (Prestrud 1991; VanderWall and Jenkins 2003) and birds (Bugnyar and Kotrschal 2002; Emery and Clayton 2001), as a type of appetitive behaviour characterized by foraging and carrying food from the source to a hidden place for a period before it is consumed (Schneider et al. 2013; VanderWall 1990). Such behaviour is usually induced by the need of a food supply for specific unfavourable conditions e.g. winter season, when food sources are not available or considerably limited (Vander Wall 1990). On the other hand, food hoarding can be promoted by food deprivation in some previously classified non-hoarders (Yang et al. 2011). In invertebrates, food storage may be induced by a period of starvation, as in decapod crustaceans (Kim 2010; Wickins et al. 1996), or by specific life traits as in leafcutter ants (Mueller et al. 2011) or spiders (de Crespigny et al. 2001). But burying or caching in invertebrates is at least very sporadic and any report of exuviae caching has never been reported in invertebrates.

During acclimation of two crayfish species (representatives of both northern and southern hemisphere crayfish species) prior to experimental work unexpected evidence was found of undescribed behaviour in invertebrates. Hence, the observed caching behaviour was explored fortuitously without previous hypothesis or expectations. Present work therefore should outline the new hypothesis and challenges in research of invertebrate behavioural patterns.

## Methods

Two species were involved in the study; signal crayfish (*Pacifastacus leniusculus* (Dana, 1852), family Astacidae) and yabby (*Cherax destructor* Clark, 1936, family Parastacidae). Signal crayfish specimens were captured in a pond near Velké Meziříčí (Czech Republic; 49°22′42”N, 16°4′53”E) in April 2015. They were placed in a flow-through system with natural ambient light and water temperature conditions. Seventy specimens (young adults and sub-adults; carapace length, CL = 29.9 ± 2.5 mm, weight, w = 7.5 ± 2.0 g) were taken for acclimation for a planned study under experimental conditions (28 May 2015). Crayfish were individually stocked into plastic boxes filled with a sandy layer (200 ml–376 g of sand) and 2000 ml of tempered tap water, and placed to an incubator maintaining the water temperature at 16 °C. Five (CL = 28.3 ± 2.7 mm, w = 6.4 ± 1.7 g) of 70 stocked specimens moulted during the 24 h acclimation period. All of these exhibited exuvial burying behaviour and were therefore observed for a further 3 days at 24 h intervals (to prevent disturbance). The time from burying to exhuming of exuviae and time of its consumption was monitored.

Yabby specimens originated from our own aquarium culture reared under stable conditions of approximately 20 °C of water temperature and a light regime of 12 h light and 12 h dark. The first evidence of burying behaviour in yabby, seen in non-standardized conditions of small aquaria with sandy substrata, was discounted as a random event in March 2015. Following similar observations on signal crayfish, ten yabby specimens (CL = 25.9 ± 3.0 mm, w = 5.8 ± 2.6 g) were selected, exhibiting signs of forthcoming moulting (softened carapace) and placed in the same standardized conditions (except for water temperature, which was maintained at 20 °C) as described above for signal crayfish for 24 h. Only two crayfish (CL = 23.7 ± 1.0 mm, w = 4.4 ± 1.0 g) moulted, but both specimens exhibited the same burying pattern.

## Results and discussion

We observed unexpected behavioural pattern, caching or burying of own exuviae, in two crayfish species: yabby (*Cherax destructor*, a southern hemisphere crayfish of the family Parastacidae) and signal crayfish (*Pacifastacus leniusculus*, a northern hemisphere crayfish of the family Astacidae) in experimental conditions. Crayfish were found to bury their exuviae after moulting event prior to its later consumption in 2–3 days. This is the first evidence of exuvial caching in invertebrates.

This unexpected behaviour is unusual and surprisingly has escaped attention despite the fact that both species are cultured in many countries of the world (Holdich et al. 2006). The postponed consumption of exuviae, as a source of minerals for carapace re-calcification, is probably because mouthparts are still hardening from internal resources during the post-moult period (Reynolds 2002). During this period lasting 2 or 3 days, when soft shelled specimens are unable to defend themselves, the exuviae are buried in suitable textured substrata. Food hoarding can be promoted by previous food deprivation in some previously classified non-hoarders (Yang et al. 2011). However, the newly described behaviour is probably connected with self-protective activities because the exuviae left beside a shelter can mark an available helpless prey.

**Fig. 1:**
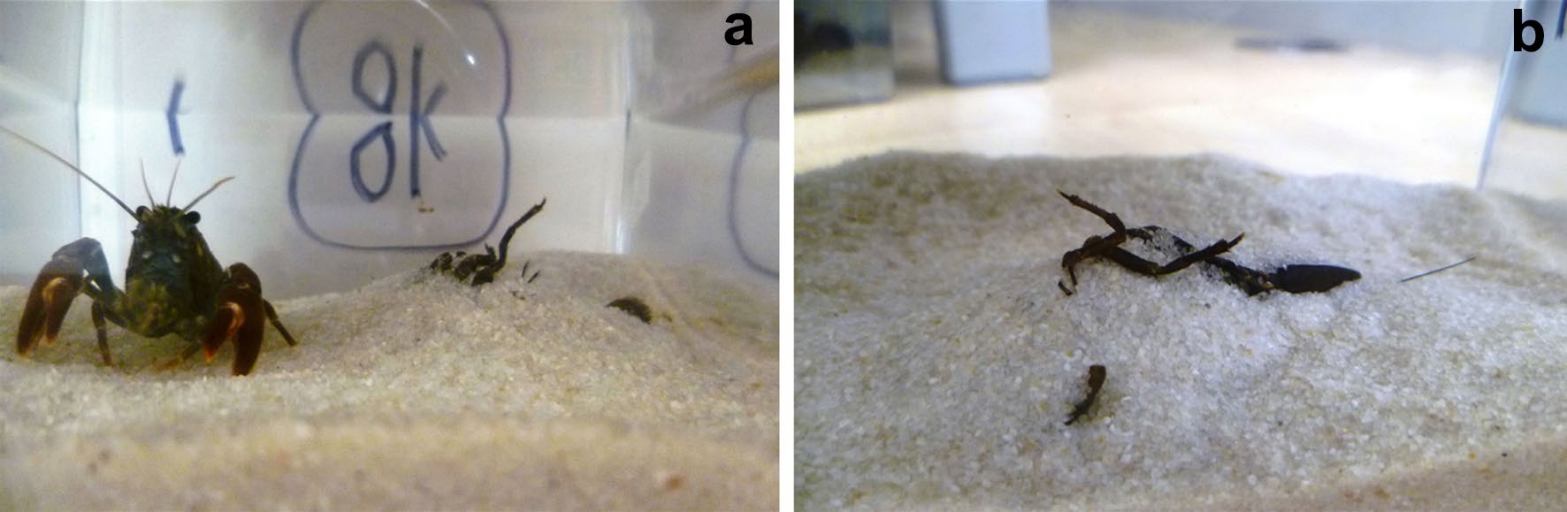
Signal crayfish (*Pacifastacus leniusculus*), initial carapace length = 32.4 mm, and its burial mound for exuviae located centrally in aquaria (**a**) and a detailed view of exuviae buried in sand (**b**)

The caching ability was not the primary reason of the research, but was observed fortuitously, when yabby and signal crayfish, were placed individually in small aquaria to acclimate for 24 h with a sandy layer prior to experiments designed for different purposes. The observed behavioural patterns have remained undetected over many decades of decapod research, probably due to many issues which could becloud or prevent the observation of this pattern (e.g inappropriate conditions for observation of such events in natural conditions, tanks and aquaria without suitable substrata, use of animals in intermoult period). Another possible reason could be marginalizing of the observed events, as we did at first case with the yabby. In that case, the burying and exhuming of exuviae was marked as an interesting but probably random event caused by digging activity. Despite this, the burying pattern was later confirmed in two other moulted yabbies. In signal crayfish, all five moulted specimens independently buried their exuviae under a mound of sand (Fig. 1), which is not a sign of a random phenomenon. The last reason why this behavioural pattern may have been overlooked is the likely frequent failure of the burying activity, perhaps in response to the presence of potential danger, which can frequently interrupt caching (Bugnyar and Kotrschal 2002).

**Fig. 2:**
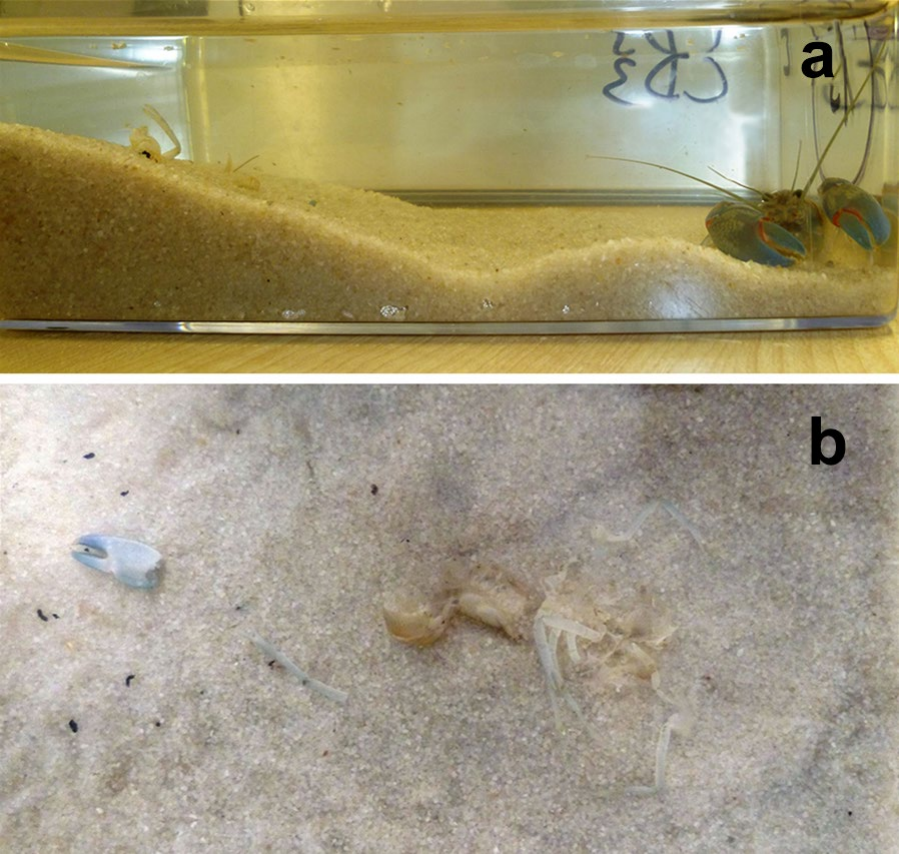
The burial mound of yabby (*Cherax destructor*), initial carapace length = 24.7 mm, made by heaping sand against the side of the aquarium (**a**) and its excavation during consumption of the exuviae (**b**)

All observed specimens exhumed and ate their exuviae after 2 or 3 days (Fig. 2). The delay between moulting and eating exuviae cannot be marked as hoarding, because that behaviour is directly connected with food (VanderWall 1990), whereas exuviae are not a main or the only food item, despite the fact that it can be utilized as valuable source of minerals for recalcification of exoskeleton (Reynolds 2002). A better designation is caching or, even better, burying, because the event is only completed by burial in a sandy mound.

The explicit reason for such behaviour is unknown, but may be motivated by two main reasons. Firstly, hiding and storing of this easily available source of minerals for re-calcification [internal resources are insufficient to harden the whole exoskeleton (Reynolds 2002)] during the time when crucial feeding structures such as chelae mouthparts and other limbs are hardening from internal resources (particularly from gastroliths) (Greenaway 1985). After this period, mouthparts are able to chew and exuviae can be uncovered and eaten. Secondly, by burying their exuviae, just moulted crayfish hide their own soft body vulnerability, as considerable mortality occurs by predation or cannibalism in this time (Reynolds 2002). Exuviae are hidden from the sight of both conspecifics and heterospecifics and their concealment through burial can limit location of freshly moulted specimens. It must be said that crayfish are unable to guard the hiding place of their exuviae and pilferage can often occur. On the other hand, such pilferage leads only to resource loss, while the builder of the burial mound remains unseen.

## Conclusions

Generally, the described type of caching has not been classified among the known types of hoarding or caching described in other animal taxa (Dally et al. 2006; VanderWall 1990). Nevertheless, the controlling mechanisms of such behaviour could be similar and can suggest broader and more complex behavioural patterns in invertebrates so far unknown. Mechanisms inducing or inhibiting the newly observed behaviour remain unidentified, and represent a new challenge for future research as well as presence/absence of this behavioural patterns in other decapods. Present work therefore should outline the new hypothesis and challenges in research of invertebrate behavioural patterns.
